# The PTI‐suppressing Avr2 effector from *Fusarium oxysporum* suppresses mono‐ubiquitination and plasma membrane dissociation of BIK1

**DOI:** 10.1111/mpp.13369

**Published:** 2023-06-30

**Authors:** Mila C. Blekemolen, Zunyong Liu, Martin Stegman, Cyril Zipfel, Libo Shan, Frank L. W. Takken

**Affiliations:** ^1^ Molecular Plant Pathology, Swammerdam Institute of Life Science University of Amsterdam Amsterdam Netherlands; ^2^ Department of Biochemistry & Biophysics Texas A&M University College Station Texas USA; ^3^ The Sainsbury Laboratory University of East Anglia Norwich UK; ^4^ Institute of Plant and Microbial Biology, Zurich‐Basel Plant Science Center University of Zurich Zurich Switzerland; ^5^ Present address: Phytopathology, School of Life Sciences Technical University of Munich Freising Germany

**Keywords:** defence signalling, effectors, *Fusarium*, plant immunity, receptor‐like cytoplasmic kinases

## Abstract

Plant pathogens use effector proteins to target host processes involved in pathogen perception, immune signalling, or defence outputs. Unlike foliar pathogens, it is poorly understood how root‐invading pathogens suppress immunity. The Avr2 effector from the tomato root‐ and xylem‐colonizing pathogen *Fusarium oxysporum* suppresses immune signalling induced by various pathogen‐associated molecular patterns (PAMPs). It is unknown how Avr2 targets the immune system. Transgenic *AVR2 Arabidopsis thaliana* phenocopies mutants in which the pattern recognition receptor (PRR) co‐receptor BRI1‐ASSOCIATED RECEPTOR KINASE (BAK1) or its downstream signalling kinase BOTRYTIS‐INDUCED KINASE 1 (BIK1) are knocked out. We therefore tested whether these kinases are Avr2 targets. Flg22‐induced complex formation of the PRR FLAGELLIN SENSITIVE 2 and BAK1 occurred in the presence and absence of Avr2, indicating that Avr2 does not affect BAK1 function or PRR complex formation. Bimolecular fluorescence complementation assays showed that Avr2 and BIK1 co‐localize in planta. Although Avr2 did not affect flg22‐induced BIK1 phosphorylation, mono‐ubiquitination was compromised. Furthermore, Avr2 affected BIK1 abundance and shifted its localization from nucleocytoplasmic to the cell periphery/plasma membrane. Together, these data imply that Avr2 may retain BIK1 at the plasma membrane, thereby suppressing its ability to activate immune signalling. Because mono‐ubiquitination of BIK1 is required for its internalization, interference with this process by Avr2 could provide a mechanistic explanation for the compromised BIK1 mobility upon flg22 treatment. The identification of BIK1 as an effector target of a root‐invading vascular pathogen identifies this kinase as a conserved signalling component for both root and shoot immunity.

## Graphical abstract

Avr2 from *Fusarium oxysporum* co‐localizes with BIK1 and affects flg22‐induced mono‐ubiquitination, membrane dissociation and protein accumulation, but not phosphorylation of the signalling kinase.

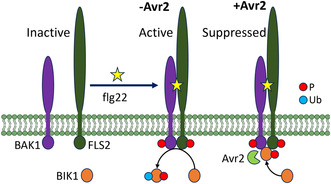

## 1 INTRODUCTION

Plant roots lack the outermost cuticle layer that is present in aboveground structures, that is, leaves or stems. Without this protective barrier, the outer cell layers of the roots are more easily accessible to microbes (De Coninck et al., 2015). Because the microbe‐rich soil environment of roots is composed of both beneficial and pathogenic microbes, the activation of root defence responses must be able to distinguish friend from foe (Hacquard et al., 2017; Zhou et al., 2020). The immune pathways in aboveground structures are more accessible for research and therefore more thoroughly researched than the defences present in roots (De Coninck et al., 2015). It is for this reason that our current understanding of the plant immune system is mostly based on the recognition of foliar pathogens (Couto & Zipfel, 2016; Saijo et al., 2018; Tang et al., 2017). Therefore, it is important to expand our knowledge on resistance mechanisms acting in root tissues and study how root‐infecting pathogens manipulate root‐based immunity.

Plants have evolved a two‐layered immune system to detect biotic threats (e.g., bacteria, fungi, oomycetes) and prevent disease. Recognition of conserved microbe‐derived molecules or pathogen‐associated molecular patterns (PAMPs) by cell surface immunity receptors (pattern recognition receptors [PRR]) activates a generic defence response called pattern‐triggered immunity (PTI) (Couto & Zipfel, 2016; Saijo et al., 2018; Tang et al., 2017). PTI responses include several outputs, ranging from early responses, for example, reactive oxygen species (ROS) production, MITOGEN‐ACTIVATED PROTEIN KINASE (MAPK) activation and transcriptional reprogramming, to late responses such as callose deposition and growth reduction (Couto & Zipfel, 2016; Saijo et al., 2018; Tang et al., 2017). Bacterial flagellin (and its derivative, the flg22 peptide) and fungal chitin are two well‐studied PAMPs that are recognized by *Arabidopsis thaliana* PRRs FLAGELLIN SENSING 2 (FLS2) and CHITIN ELICITOR RECEPTOR KINASE 1 (CERK1)/LYSINE MOTIF RECEPTOR KINASE 5 (LYK5), respectively. On flg22 binding, FLS2 forms a signalling complex with its co‐receptor BAK1. BAK1, also named SOMATIC EMBRYOGENENIS RECEPTOR KINASE 3 (SERK3), is a conserved member of the SERK family of leucine–rich repeat receptor–like kinases. BAK1 is also known to associate with other PRRs, namely EF‐TU RECEPTOR (EFR), PEP RECEPTOR 1/2 (PEPR1/2) and RECEPTOR‐LIKE PROTEIN 23 (RLP23), to form signalling complexes (Yasuda et al., 2017). The activation of specific defence outputs is often carried out by receptor‐like cytoplasmic kinases (RLCKs) downstream of PAMP recognition by PRRs. Many RLCKs belong to the extensive PBS1‐LIKE KINASE (PBL) protein family. The RLCK BIK1 is involved in defence regulation downstream of many PRR complexes (FLS2–BAK1, CERK1–LYK5, EFR and PEPR1/2–BAK1) (Couto & Zipfel, 2016). On flg22 perception by the FLS2–BAK1 complex, BAK1 phosphorylates BIK1 at multiple sites (Lin et al., 2014; Lu et al., 2010). Subsequently, BIK1 is mono‐ubiquitinated by the E3 ligases RING‐H2 FINGER A3A (RHA3A) and RHA3B, allowing it to dissociate from the FLS2–BAK1 complex and activate defence responses such as ROS accumulation, callose deposition and defence gene activation (Ma et al., 2020). It does so by, for example, interacting with and phosphorylating the ROS–generating RESPIRATORY BURST OXIDASE HOMOLOGUE D (RBOHD) complex in the plasma membrane (PM) or interacting with WRKY transcription factors in the nucleus (Kadota et al., 2014; Lal et al., 2018; Li et al., 2014).

The second layer of plant immunity relies on recognition of pathogen‐produced virulence factors, called effectors, by either intracellular nucleotide‐binding leucine‐rich repeat (NB‐LRR) or cell‐surface receptor‐like kinase (RLK)/receptor‐like protein (RLP)‐type receptors. Effector recognition by these resistance (R) proteins induces a strong defence response, often culminating in localized cell death and hence referred to as the hypersensitive response (HR) (Jones & Dangl, 2006). Elucidating the host processes targeted by effector proteins is important to understand the virulence strategy of a pathogen and the basis of host susceptibility. Bacterial effectors are known to manipulate PTI at different signalling levels, that is, at PRRs, co‐receptors, RLCKs, or further downstream, targeting the MAPK pathways or WRKY transcription factors (Dou & Zhou, 2012). *Pseudomonas syringae* effector AvrPto directly targets the PRR complexes FLS2, EFR, and BAK1, blocking PTI probably through inhibition of the PRR kinase activity and complex formation (Shan et al., 2008; Xiang et al., 2008; Xing et al., 2007). Another *P. syringae* effector, AvrPtoB, interacts with co‐receptor BAK1 through its kinase binding domain (Cheng et al., 2011). Multiple members of the PBL family of RLCKs (including BIK1) are targeted by *P. syringae* effector AvrPphB via protease cleavage (Zhang et al., 2010). The MAPK pathway is a more downstream signalling process that is often targeted. For example, *P. syringae* effector HopAI1 is a phosphothreonine lyase that dephosphorylates MAP KINASE KINASE 5 (MKK5), disrupting the MAPK cascade (Wang et al., 2010). Fungal effectors tend to employ different immune disruption tactics (e.g., interference with PAMP perception, manipulation of metabolic processes or transcriptional regulators) (Buscaill & van der Hoorn, 2021; Djamei et al., 2011; Lo Presti et al., 2015; Tanaka et al., 2014). *Magnaporthe oryzae* effector NIS1 is one of the few examples of a fungal effector suppressing PTI by targeting both BAK1 and BIK1 (Irieda et al., 2019). Currently, it is poorly understood whether root‐invading pathogens use similar mechanisms to suppress plant defence as those employed by their foliar counterparts.

The soilborne pathogen *Fusarium oxysporum* is a widespread root colonizer. Colonization of the root surface and cortex is often symptomless, but pathogenic isolates can invade the vasculature. The latter eventually leads to blockage of the xylem vessels, causing vascular wilt disease in a wide variety of plant species (Michielse & Rep, 2009). Each *F. oxysporum forma specialis* (f. sp.) has its unique host. *F. oxysporum* f. sp. *lycopersici* (Fol) infects tomato; it directs itself toward the root surface via peroxidase sensing, where it enters the root system through cracks and wounds (Nordzieke et al., 2019). From the point of entry, fungal hyphae spread throughout the apoplast to eventually colonize the vasculature (de Lamo & Takken, 2020). Fol effector proteins are secreted in apoplastic spaces of the root cortex and in the xylem sap during infection. Fol‐secreted effector proteins were originally identified as Secreted In Xylem (Six) proteins. Four out of 14 of these Six proteins (Six1, Avr2 [Six3], Six5, and Six6) have been shown to be required for full Fol pathogenicity (Gawehns et al., 2014; Houterman et al., 2009; Ma et al., 2015; Rep et al., 2004). Six3 was renamed Avr2 as the effector is recognized by the NB‐LRR R protein I‐2 (Houterman et al., 2009). As a virulence factor, Avr2 suppresses several PTI responses, including ROS accumulation, callose deposition and MAPK activation, on flg22, chitin, chitosan and NLP (necrosis and ethylene‐inducing protein; nlp24) treatment (Coleman et al., 2021; de Lamo et al., 2021; Di, Cao, et al., 2017; Tintor et al., 2020). However, it is currently unknown which PTI signalling protein(s) are targeted by Avr2 to suppress these defence responses. The crystal structure of Avr2 shows a β‐barrel conformation that shares structural homology with ToxA from *Pyrenophora tritici‐repentis* and with Tumour necrosis factor Receptor Associated Factor (TRAF) domain–containing proteins (Di, Cao, et al., 2017). TRAF proteins, often acting as cytosolic adaptor proteins in mammals, regulate NLR turnover in *A. thaliana* by interacting with E3 ligase complexes modulating substrate ubiquitination (Huang et al., 2016). Because several PTI outputs, triggered by highly diverse PAMPs, are affected by Avr2, it seems unlikely that a (single) PRR would be an Avr2 target. Of note, an *Arabidopsis thaliana bak1‐5* mutant shows a reduction in flg22‐ and elf18‐induced ROS accumulation, MAPK activity and defence gene expression, while retaining wild‐type‐like development and morphology (Schwessinger et al., 2011). The *A. thaliana* loss‐of‐function *bik1* mutant shows a reduction in flg22‐, elf18‐, and chitin‐induced ROS accumulation, callose deposition, and a reduced growth phenotype (Zhang et al., 2010), mimicking the phenotype of transgenic Avr2 plants (Di, Cao, et al., 2017). The overlap between defence outputs altered by Avr2 and *bak1* and *bik1* mutants suggests BAK1 and/or BIK1 as candidates for Avr2‐mediated PTI suppression.

We therefore investigated whether co‐receptor BAK1 and/or RLCK BIK1 are affected by Avr2, by studying (i) formation of the FLS2–BAK1–BIK1 complex on flg22 application in the absence and presence of Avr2, (ii) the protein accumulation, phosphorylation, and ubiquitination state of these PTI signalling proteins, (iii) the proximity and potential interaction between Avr2 and BIK1, and (iv) the intracellular localization pattern of BIK1 in the presence of Avr2 wild‐type or loss‐of‐virulence mutants. We conclude that Avr2 interferes with PAMP‐induced mono‐ubiquitination of BIK1 and observe retention of this RLCK at the PM corresponding with its compromised ability to induce PTI responses.

## 2 RESULTS

### 2.1 Avr2 does not affect heterodimerization of the FLS2–BAK1 immune signalling complex on flg22 application

**FIGURE 1:**
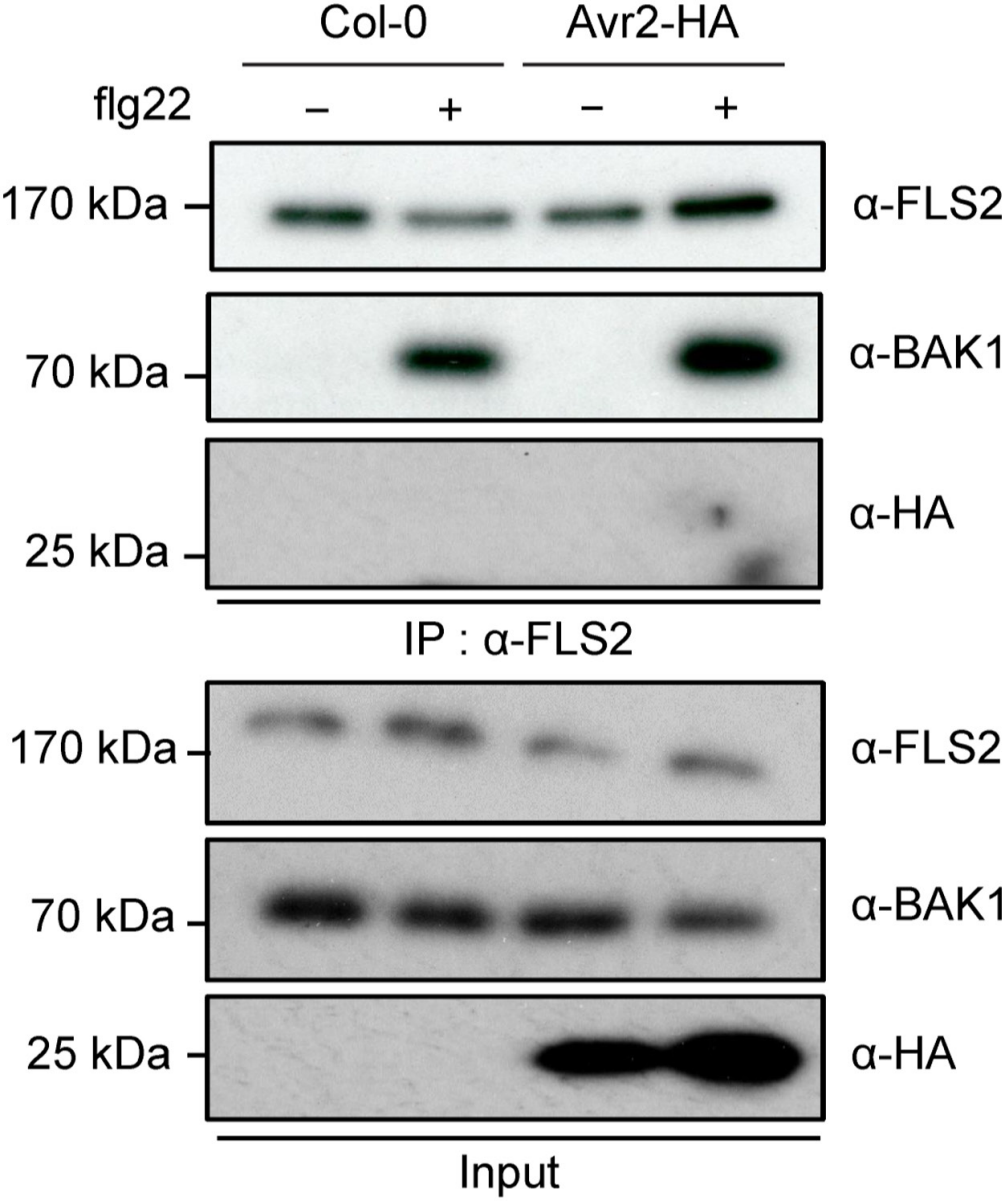
Avr2 does not affect heterodimerization of the FLS2–BAK1 immunocomplex on flg22 application. Immunoblot depicting FLAGELLIN SENSING 2 (FLS2) and BRI1‐ASSOCIATED RECEPTOR KINASE (BAK1) accumulation in wild‐type *Arabidopsis thaliana* Col‐0 or transgenic Δ*spAvr2* expressing seedlings on water or flg22 treatment (100 nM). Ten minutes after treatment plant proteins were extracted and separated using SDS‐PAGE and subsequently immunoblotted. Avr2, FLS2, and BAK1 protein levels were visualized using anti‐HA, anti‐FLS2, and anti‐BAK1 antibodies, respectively. After immunoprecipitation (IP) with anti‐FLS2 agarose beads, FLS2 and BAK1 were detected by immunoblot using anti‐FLS2 and anti‐BAK1 antibodies. Molecular weight markers are indicated on the left.

Flg22‐triggered FLS2 signalling is suppressed by Avr2 (Di, Cao, et al., 2017). Because the binding of flg22 by FLS2 prompts the recruitment of BAK1 (Chinchilla et al., 2007; Heese et al., 2007; Schulze et al., 2010; Sun et al., 2022), we investigated whether this recruitment process is disrupted in the presence of Avr2. Immunoprecipitation assays were performed on protein extracts isolated from seedlings of wild‐type (Col‐0) or transgenic Δ*spAvr2 A. thaliana*. To ensure a cytosolic localization of the effector protein, the region encoding its signal peptide was omitted (Δsp). Accumulation of the endogenous FLS2 and BAK1 proteins was confirmed in protein extracts from both Col‐0 and ΔspAvr2 plants using anti‐FLS2 and anti‐BAK1 antibodies, respectively (Figure 1, input). As expected, HA‐tagged Avr2 was detected solely in ΔspAvr2 plants. On anti‐FLS2 immunoprecipitation, BAK1 co‐precipitation was observed on flg22 treatment in both Col‐0 and ΔspAvr2 plants. No co‐precipitation was observed in the mock treatment (Figure 1, IP). These data show that heterodimerization of FLS2 and BAK1 was not impaired by Avr2. Avr2‐HA did not co‐precipitate with the FLS2–BAK1 complex, suggesting that it does not directly interact with these proteins.

### 2.2 Avr2 and BIK1 co‐localize in planta

Because heterodimerization of BAK1 with FLS2 was not affected by Avr2, we investigated whether Avr2 acts on downstream signalling components of the receptor complex. The RLCK BIK1 is a known signalling hub involved in FLS2/BAK1– and LYK5/CERK1–regulated defence (Couto & Zipfel, 2016; Lu et al., 2010; Zhang et al., 2010). Given the ability of Avr2 to suppress flg22‐, chitin‐, and nlp24‐triggered PTI responses (Coleman et al., 2021; de Lamo & Takken, 2020; Di, Cao, et al., 2017; Tintor et al., 2020), BIK1 could be a target for Avr2 to disrupt PTI signalling.

**FIGURE 2:**
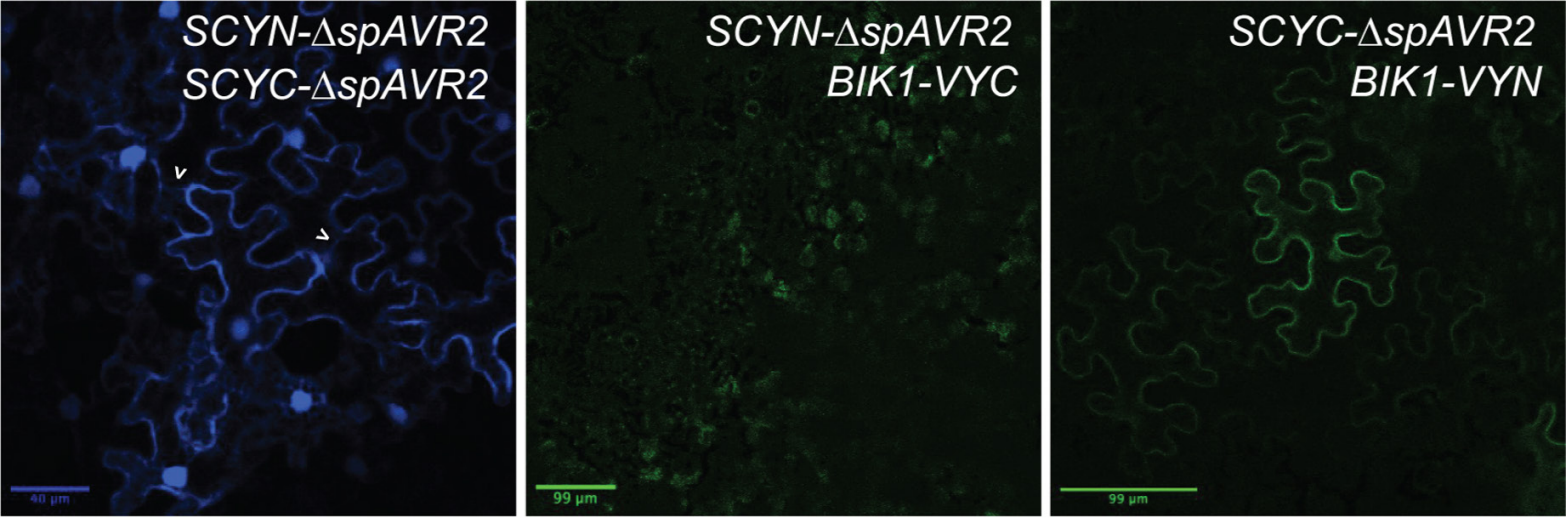
Avr2 and BIK1 co‐localize in planta. Confocal microscopy images of agro‐infiltrated *Nicotiana benthamiana* leaves transiently expressing bimolecular fluorescence complementation constructs (*SCYC‐*Δ*spAVR2* and *SCYN‐*Δ*spAVR2*, *BIK1‐VYN* and *SCYC‐*Δ*spAVR2*, or *BIK1‐VYC* and *SCYN‐*Δ*spAVR2*). Cyan and green fluorescence represent protein–protein interactions visualized by complementation of the fluorescent protein halves. Green and blue fluorescence was analysed 48 h after agro‐infiltration by confocal microscopy. Three biological replicates were performed. Scale bars represent 40 or 99 μm.

To study whether Avr2 co‐localizes with BIK1, bimolecular fluorescence complementation (BiFC) assays were performed to assess, in planta, the potential proximity of the two proteins. Constructs were generated encoding *A. thaliana* BIK1 C‐terminally fused to either the N‐terminal half or the C‐terminal half of the VENUS fluorescent protein (VYN or VYC) (Gehl et al., 2009). In addition, two constructs were generated encoding ΔspAvr2 N‐terminally fused to either the N‐ or C‐terminal half of the SUPERCYAN fluorophore (SCYN or SCYC) (Gehl et al., 2009). Co‐expression of the BiFC constructs in *Nicotiana benthamiana* leaves was performed via agro‐infiltration and blue/green fluorescence, indicating potential protein–protein interactions, was analysed using confocal microscopy. Given the ability of Avr2 to form homodimers, *SCYN*‐Δ*spAvr2* and *SCYC*‐Δ*spAvr2* were co‐expressed as positive controls. As expected, a strong blue nucleocytoplasmic fluorescence was observed for the latter constructs, confirming Avr2 dimerization (Figure 2, left panel). In cells co‐expressing *SCYN‐*Δ*spAvr2* and *BIK1‐VYC* no green fluorescence could be detected (Figure 2, middle panel). However, the *SCYC*‐Δ*spAvr2* and *BIK1VYN* combination did result in a bright‐green fluorescent signal. This signal was mostly located at the cell periphery but was also weakly visible in the nucleus (Figure 2, right panel). Notably, fluorescence originating from the proposed SCYC–ΔspAvr2 and BIK1–VYN interaction was not observed in cytosolic strands, as opposed to the SCYN–ΔspAvr2 and SCYC–ΔspAvr2 interaction, where cytosolic strands were clearly detectible (Figure 2, left and right panels, indicated by arrows; Figure S1). To investigate whether the proteins associate, co‐immunoprecipitation assays were performed in *A. thaliana* Col‐0 protoplast transfected with constructs encoding HA‐tagged BIK1 and FLAG‐tagged ΔspAvr2. Although both proteins were readily detectable in the input material and Avr2 could be successfully pulled down using the HA tag, no co‐immunoprecipitation of HA‐BIK1 was observed (Figure S2). Taken together, the fluorescence complementation indicates proximity of the Avr2 and BIK1 proteins mostly at the cell periphery.

### 2.3 Avr2 mutants that do not suppress PTI remain co‐localized with BIK1

**FIGURE 3:**
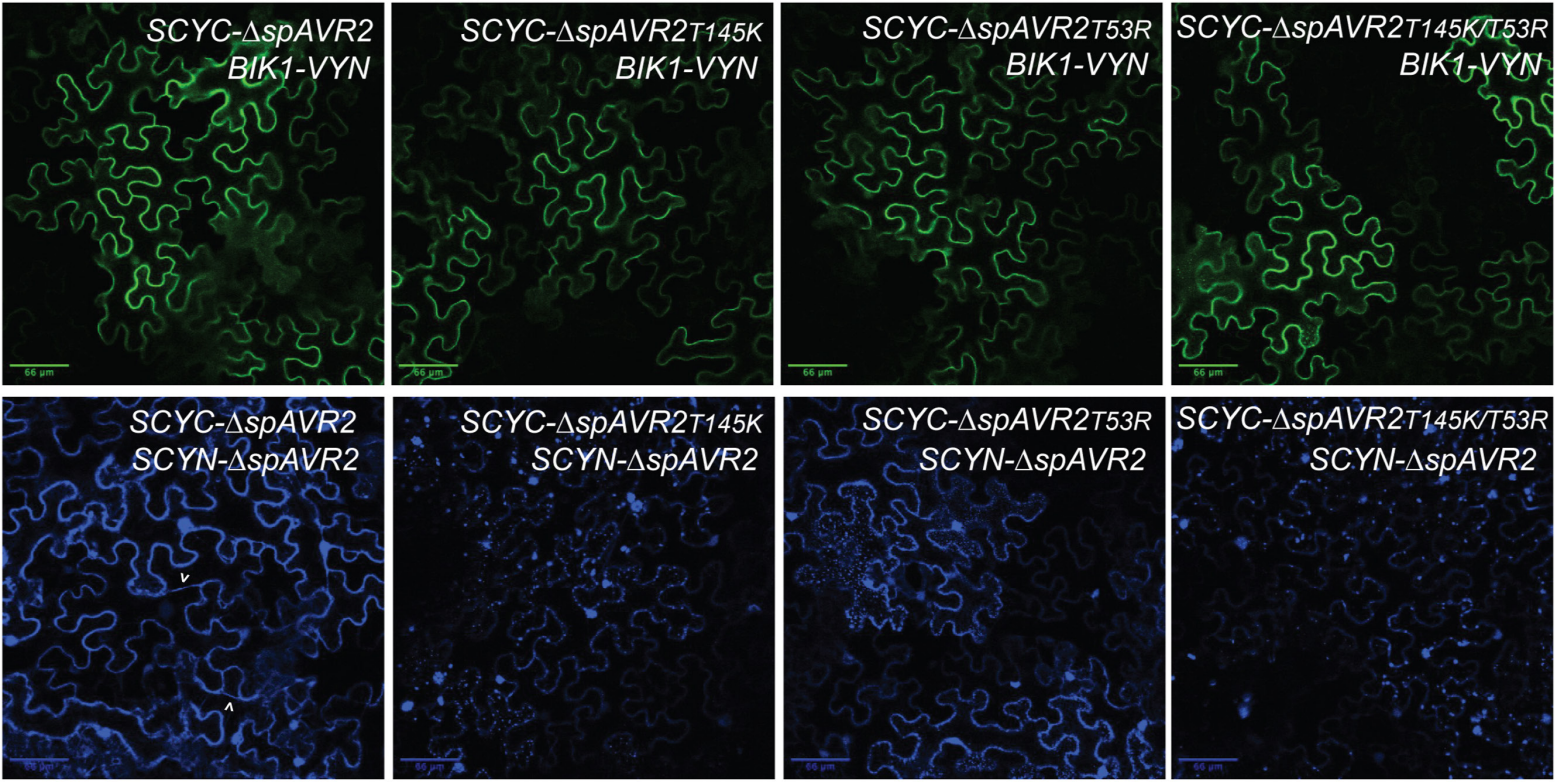
Avr2 mutants that do not suppress PTI do co‐localize with BIK1. Confocal microscopy images of agro‐infiltrated *Nicotiana benthamiana* leaves transiently expressing bimolecular fluorescence complementation construct *BIK1‐VYN* with *SCYC‐*Δ*spAVR2*, *SCYC‐*Δ*spAVR2T145K*, *SCYC‐*Δ*spAVR2T53R*, or *SCYC‐*Δ*spAVR2T145K/T53R*, and *SCYN‐*Δ*spAVR2* with *SCYC‐*Δ*spAVR2*, *SCYC‐*Δ*spAVR2T145K*, *SCYC‐*Δ*spAVR2T53R*, and *SCYC‐*Δ*spAVR2T145K/T53R*. Cyan and green fluorescence represent protein–protein interactions via complementation of the fluorescent protein halves. Green and blue fluorescence was analysed 48 h after agro‐infiltration using confocal microscopy. Three biological replicates were performed. Scale bars represent 66 μm.

Two mutations (T53R and T145K) in Avr2 have been identified that compromise the ability of the effector protein to suppress PTI but retain its *I‐2*‐mediated recognition (Di, Cao, et al., 2017). We tested whether the inability of the double and single Avr2 mutants to suppress PTI correlates with an altered co‐localization with BIK1. To study the interaction between BIK1 and the Avr2 mutants (Avr2T53R, Avr2T145K, and Avr2T145K/T53R) BiFC assays were performed. The three Avr2 variants were fused N‐terminally to SCYC (Gehl et al., 2009) to create SCYC‐Δ*spAvr2T53*, SCYC‐Δ*spAvr2T145K*, and SCYC‐Δ*spAvr2T145K/T53R*, respectively. The *SCYC*‐Δ*spAvr2* mutant and *SCYC‐*Δ*spAvr2* wild‐type BiFC constructs were co‐expressed with *BIK1‐VYN* or *SCYN*‐Δ*spAvr2* in *N. benthamiana* leaves via agro‐infiltration. Fluorescence, indicating potential protein–protein interactions, was analysed using confocal microscopy. As before, strong green fluorescence at the cell periphery and a weak nuclear signal was observed in cells expressing *BIK1‐VYN* and *SCYC*‐Δ*spAvr2* (Figure 3, upper left panel), reaffirming the proximity between Avr2 and BIK1. Of note, co‐expression of *BIK1‐VYN* with *SCYC*‐Δ*spAvr2T53*, *SCYC*‐Δ*spAvr2T145K*, or *SCYC*‐Δ*spAvr2T145K/T53R* similarly resulted in green fluorescence at the cell periphery and a weak nuclear signal (Figure 3, upper middle/right panels). These data indicate proximity between the Avr2 mutants and BIK1. Additionally, we monitored the ability of wild‐type Avr2 to dimerize with Avr2 mutants. In cells co‐expressing wild‐type *SCYC*‐Δ*spAvr2* and *SCYN*‐Δ*spAvr2*, strong blue fluorescence was observed in the nucleus, in cytosolic strands (indicated by arrows), and at the cell periphery (Figure 3, lower left panel), confirming Avr2 dimerization. However, on co‐expression of *SCYN*‐Δ*spAvr2* with *SCYC*‐Δ*spAvr2T53*, *SCYC*‐Δ*spAvr2T145K*, or *SCYC*‐Δ*spAvr2T145K/T53R* a much weaker cytosolic fluorescence was observed, in addition to relatively large fluorescent punctate structures. These structures were located mostly at the cell periphery and their heterogenous size implies that they are aggregates (Figure 3, lower middle/right panels). Altogether, the point mutations in Avr2 did not result in loss of co‐localization with BIK1 but they did disrupt Avr2 homodimerization, resulting in the formation of protein aggregates.

### 2.4 Avr2 differentially affects BIK1 accumulation in *N. benthamiana* and *A. thaliana*

**FIGURE 4:**
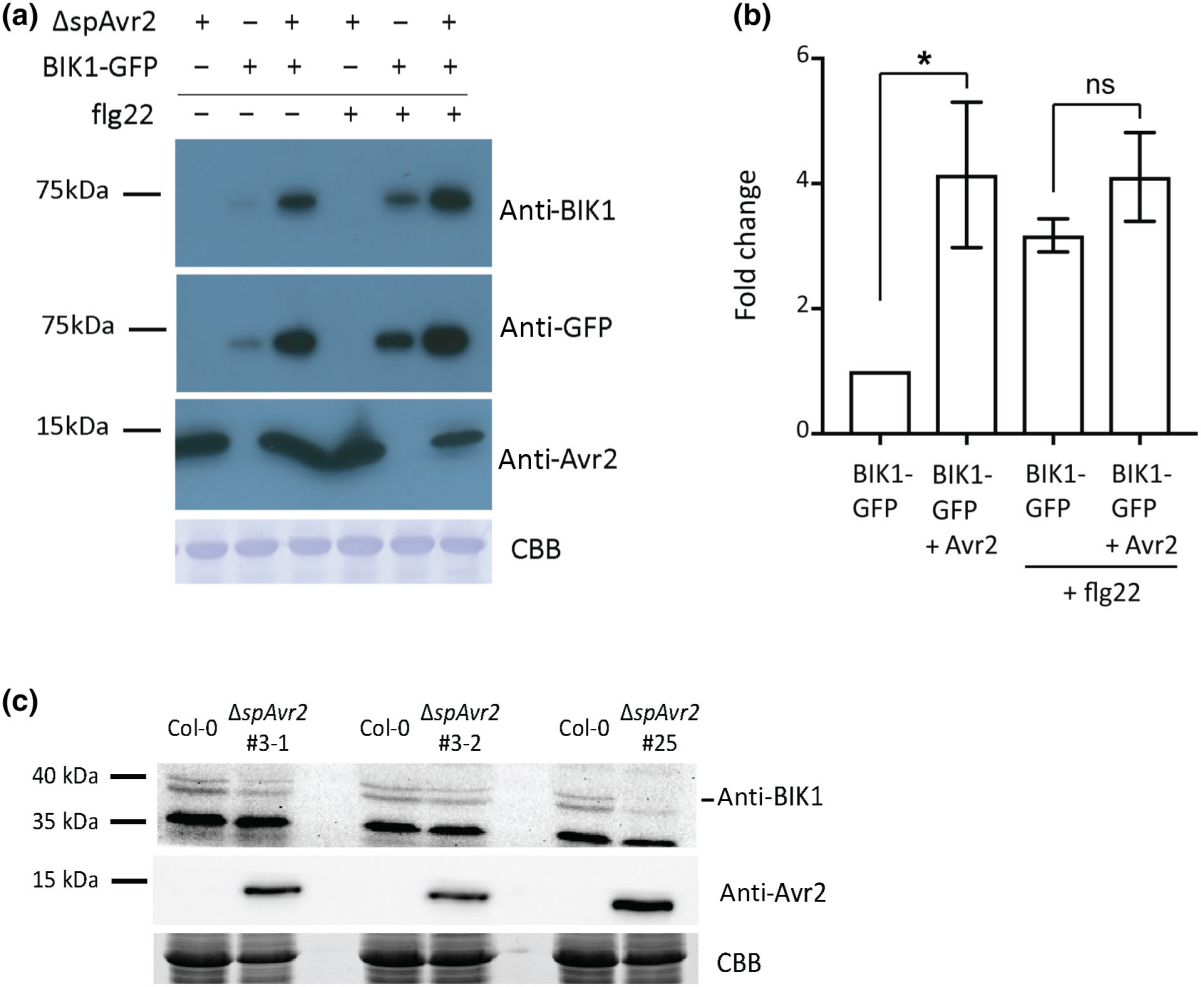
BOTRYTIS‐INDUCED KINASE 1 (BIK1) accumulation is affected by the presence of Avr2. (a) Immunoblot depicting BIK1 protein accumulation in the presence or absence of Avr2 in *Nicotiana benthamiana*. *BIK1‐GFP* and/or Δ*spAVR2* were (co‐)expressed in *N. benthamiana* via agro‐infiltration and after 48 h the infiltrated leaf sectors were treated with either water or flg22 (100 nM) for 10 min. Total protein was isolated from leaf disks and visualized using immunoblot analysis. Avr2 protein levels were visualized using an anti‐Avr2 antibody (middle panel). BIK1 proteins levels were visualized using an anti‐BIK1 and anti‐GFP antibody (top and middle panels). Equal protein loading was verified by Coomassie brilliant blue (CBB) staining of the blot (bottom panel). Molecular weight markers are indicated on the left. (b). Quantification of BIK1 protein accumulation (depicted as ratios) by measuring band intensity of anti‐BIK1 immunoblot as area under the curve for three to five independent experiments using ImageJ. (c). Immunoblot depicting BIK1 protein accumulation in *Arabidopsis thaliana* Col‐0 and transgenic Δ*spAVR2*. Proteins were isolated from leaves and detected using immunoblot analysis. Avr2 protein levels were visualized using an anti‐Avr2 antibody (middle panel). BIK1 proteins levels were visualized using an anti‐BIK1 (top panel). Equal protein loading was verified by CBB staining of the blot (bottom panel). Molecular weight markers are indicated on the left.

Because Avr2 co‐localizes with BIK1, it is conceivable that Avr2, like other effectors (He et al., 2020), affects BIK1 accumulation. To investigate whether Avr2 targets BIK1 for degradation, we assessed the effect of Avr2 on BIK1 protein accumulation in *N. benthamiana*. Binary constructs containing either Δ*spAVR2* or *BIK1‐GFP* were (co‐)expressed via agro‐infiltration in *N. benthamiana*. Proteins were isolated from leaf disks harvested from infiltrated sectors. Immunoblots probed with anti‐BIK1 or anti‐GFP showed bands corresponding to BIK1‐GFP in both the absence and the presence of Avr2 (Figure 4a, lanes 2 and 3). Quantification of the intensity of the BIK1 bands was determined for three to five independent experiments using an ImageJ analysis tool. Notably, in the presence of Avr2, accumulation of BIK1 increased by approximately 4‐fold as compared to the mock control (Figure 4b, bars 1 and 2).

On flg22 recognition, the FLS2–BAK1 complex activates BIK1 through phosphorylation and ubiquitination, facilitating its release from the membrane‐localized complex into the cytosol. To study whether Avr2 also affects accumulation of activated BIK1, a 10‐min flg22 treatment was performed prior to leaf disk harvesting of *AVR2* and/or *BIK1‐GFP* agro‐infiltrated leaves. Using anti‐BIK1 and anti‐GFP immunoblots, an approximately 3‐fold increase in BIK1‐GFP protein abundance was observed in agro‐infiltrated leaf disks after flg22 treatment (Figure 4a, lanes 2 and 5; Figure 4b, bars 1 and 3). As the increased signal was detectable within 10 min after flg22 application, it is unlikely to be explained by up‐regulation of *BIK1* transcription and/or a reduced turnover of the protein. Of note, in the presence of Avr2, irrespective of flg22 treatment, no major differences in BIK1 signals on the immunoblot were observed (Figure 4a, lanes 3 and 6; Figure 4b, bars 2 and 4).

The effect of Avr2 on steady–state BIK1 protein accumulation in *A. thaliana* leaves was assessed in wild‐type Col‐0 and transgenic Δ*spAVR2* lines. As expected, the immunoblots probed with anti‐Avr2 only revealed an Avr2‐specific band (c.12 kDa) in the Δ*spAVR2* lines (Figure 4c). Immunoblots probed with anti‐BIK1 showed bands corresponding to endogenous BIK1 (c.44 kDa) in both Col‐0 and Avr2 lines. However, a reduction in BIK1 accumulation of 0.67, 0.64, and 0.66 of wild‐type Bik1 was observed in the three Δ*spAVR2 A. thaliana* lines as compared to the wild‐type progenitor (Figure 4c). In conclusion, Avr2 differentially affects BIK1 abundance in *N. benthamiana* and *A. thaliana*.

### 2.5 Mono‐ubiquitination of BIK1 is affected by Avr2

**FIGURE 5:**
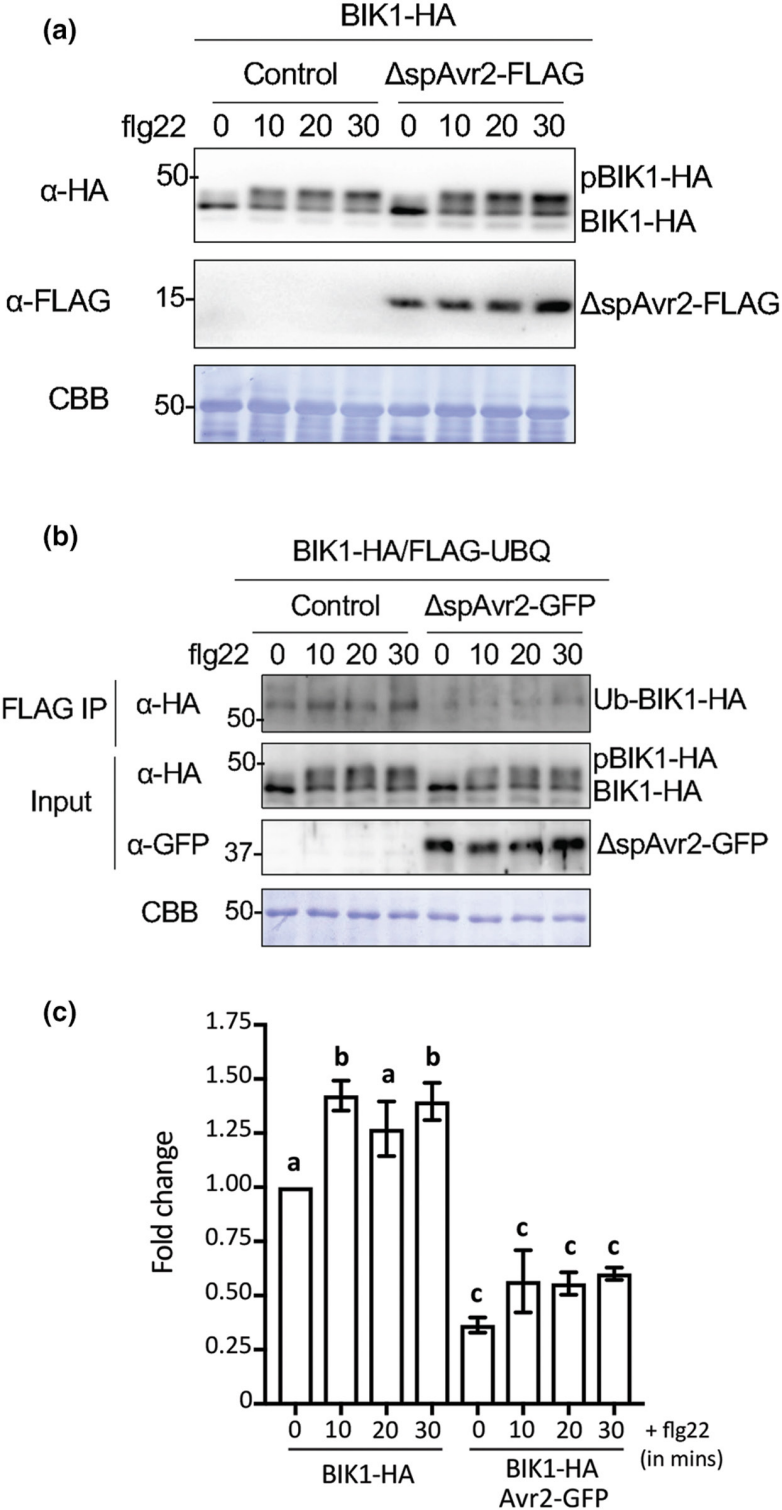
Mono‐ubiquitination of BOTRYTIS‐INDUCED KINASE 1 (BIK1) is reduced by Avr2. (a) Immunoblot depicting unphosphorylated and phosphorylated BIK1 accumulation in the presence or absence of Avr2 following flg22 treatment in *Arabidopsis thaliana* Col‐0 protoplasts. *BIK1‐HA* and/or Δ*spAVR2* were (co‐)expressed in *A. thaliana* Col‐0 protoplasts, followed by treatment with flg22 (200 nM) for 0, 5, 10, 20, or 30 min. Avr2 and BIK1 protein levels were visualized using an anti‐FLAG and anti‐HA antibodies, respectively. Equal protein loading was verified by Coomassie brilliant blue (CBB) staining of the blot. Molecular weight markers are indicated on the left. (b) Immunoblot depicting mono‐ubiquitinated BIK1 accumulation in presence or absence of Avr2 following flg22 treatment in *A. thaliana* Col‐0 protoplasts. *BIK1‐HA, FLAG‐UBQ*, and/or Δ*spAVR2‐GFP* were co‐expressed in wild‐type protoplasts, followed by treatment with flg22 (200 nM) for 0, 5, 10, 20, or 30 min. After immunoprecipitation (IP) with anti‐FLAG agarose beads, ubiquitinated BIK1 was detected by immunoblot using anti‐HA antibodies. Equal protein loading was verified by CBB staining of the blot. Molecular weight markers are indicated on the left. (c) Quantification of Ub‐BIK1 protein accumulation (depicted as ratios) by measuring band intensity of anti‐HA immunoblot as area under the curve for three independent experiments using ImageJ.

As protein accumulation of BIK1 was affected by the presence of Avr2, we set out to investigate whether Avr2 affects phosphorylation of BIK1. Transient protoplast expression assays were performed followed by immunoblot detection. HA‐tagged BIK1 (BIK1‐HA), and FLAG‐tagged ΔspAvr2 (ΔspAvr2‐FLAG) were expressed in *A. thaliana* Col‐0 protoplasts, followed by flg22 treatment for 0, 10, 20 or 30 min. At *t* = 0 BIK1‐HA was solely detected in an unphosphorylated form, as a single band of approximately 44 kDa was observed on an anti‐HA probed immunoblot (Figure 5a). On flg22 treatment, a second band of slightly higher molecular weight was observed at time points 10, 20, and 30 min, indicating an equilibrium shift toward phosphorylated BIK1 (pBIK1‐HA) (Figure 5a). In the presence of Avr2‐FLAG, which was detected using an anti‐FLAG immunoblot, a similar shift from unphosphorylated BIK1 toward phosphorylated BIK1 on flg22 treatment was observed, demonstrating that phosphorylation of BIK1 was unaffected by Avr2.

To investigate whether mono‐ubiquitination of BIK1 was affected by Avr2, in vivo ubiquitination assays were performed followed by co‐immunoprecipitation. Quantification of the intensity of the BIK1 bands was determined of three independent experiments using an ImageJ analysis tool (Figure 5c). A FLAG‐tagged ubiquitin construct (*FLAG‐UBQ*) and *BIK1‐HA* were expressed with or without Δ*spAVR2‐GFP* in *A. thaliana* Col‐0 protoplasts. On flg22 treatment, ubiquitinated proteins were pulled down with anti‐FLAG beads and the ubiquitinated BIK1‐HA (c.52 kDa) was detected on an immunoblot probed with an anti‐HA antibody (Figure 5b,c). As reported previously the amount of mono‐ubiquitinylated BIK1 in the Col‐0 protoplasts increased on flg22 treatment (Ma et al., 2020; Figure 5c). In the presence of ΔspAvr2‐GFP, mono‐ubiquitinated BIK1 could still be detected, but the bands were of lower intensity than those in the empty vector control and did not increase on flg22 treatment (Figure 5b,c). We conclude that Avr2 does not affect flg22‐induced phosphorylation of BIK1 but appears to interfere with its mono‐ubiquitination.

### 2.6 Avr2 alters the subcellular localization pattern of BIK1

**FIGURE 6:**
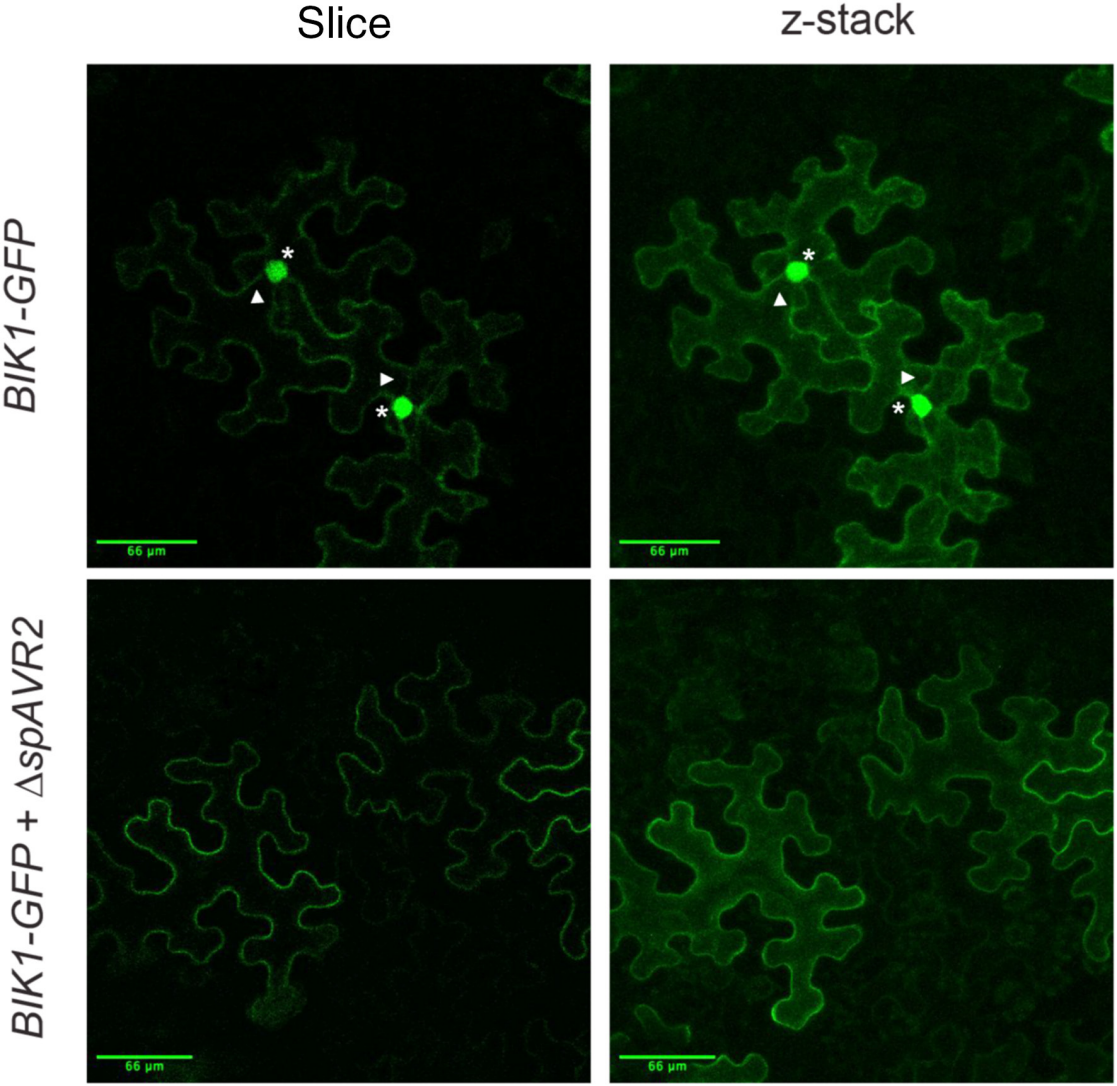
Avr2 alters the subcellular localization pattern of BOTRYTIS‐INDUCED KINASE 1 (BIK1). Confocal microscopy images of agro‐infiltrated *Nicotiana benthamiana* leaves transiently expressing *BIK1‐GFP* in the absence or presence of Δ*spAVR2*. Asterisks mark green (GFP) fluorescent signals located in the nucleus; arrowheads indicate green fluorescent signal located in cytosolic strands. Fluorescence was visualized 48 h after agro‐infiltration using confocal microscopy. Maximum projections of *z*‐stack images are depicted. Three biological replicates were performed, representative examples are shown. Scale bars represent 66 μm.

The localization of BIK1 in different subcellular compartments (i.e., the cytosol and nucleus) is important for the activation of defence responses (Kadota et al., 2014; Lal et al., 2018; Li et al., 2014). To monitor whether Avr2 affects the subcellular distribution of BIK1, a binary vector construct was generated encoding the *A. thaliana* BIK1 protein C‐terminally fused to GFP. (Co‐)expression of binary vectors carrying either *BIK1‐GFP* and/or Δ*spAvr2* through agro‐infiltration in *N. benthamiana* leaves was performed. The expected size of BIK1‐GFP (c.71 kDa) as well as the integrity of the fusion protein was confirmed using immunoblot analysis. Only minimal degradation (i.e., free GFP) was apparent, and a stronger BIK1 signal was observed in the presence of Avr2 (Figure S3). The fluorescence signal, depicting the subcellular localization pattern of BIK1‐GFP, was analysed using confocal microscopy. In the absence of Avr2, green fluorescence was observed in the nucleus, cytosolic strands, and at the cell periphery (Figure 6, top panels). However, in the presence of Avr2, green fluorescence was found predominantly at the cell periphery and not in the nucleus nor in cytosolic strands (Figure 6, bottom panels). We conclude that the subcellular localization pattern of BIK1‐GFP is altered in the presence of Avr2 such that the BIK1 protein is mostly confined to the cell periphery in the presence of the effector.

**FIGURE 7:**
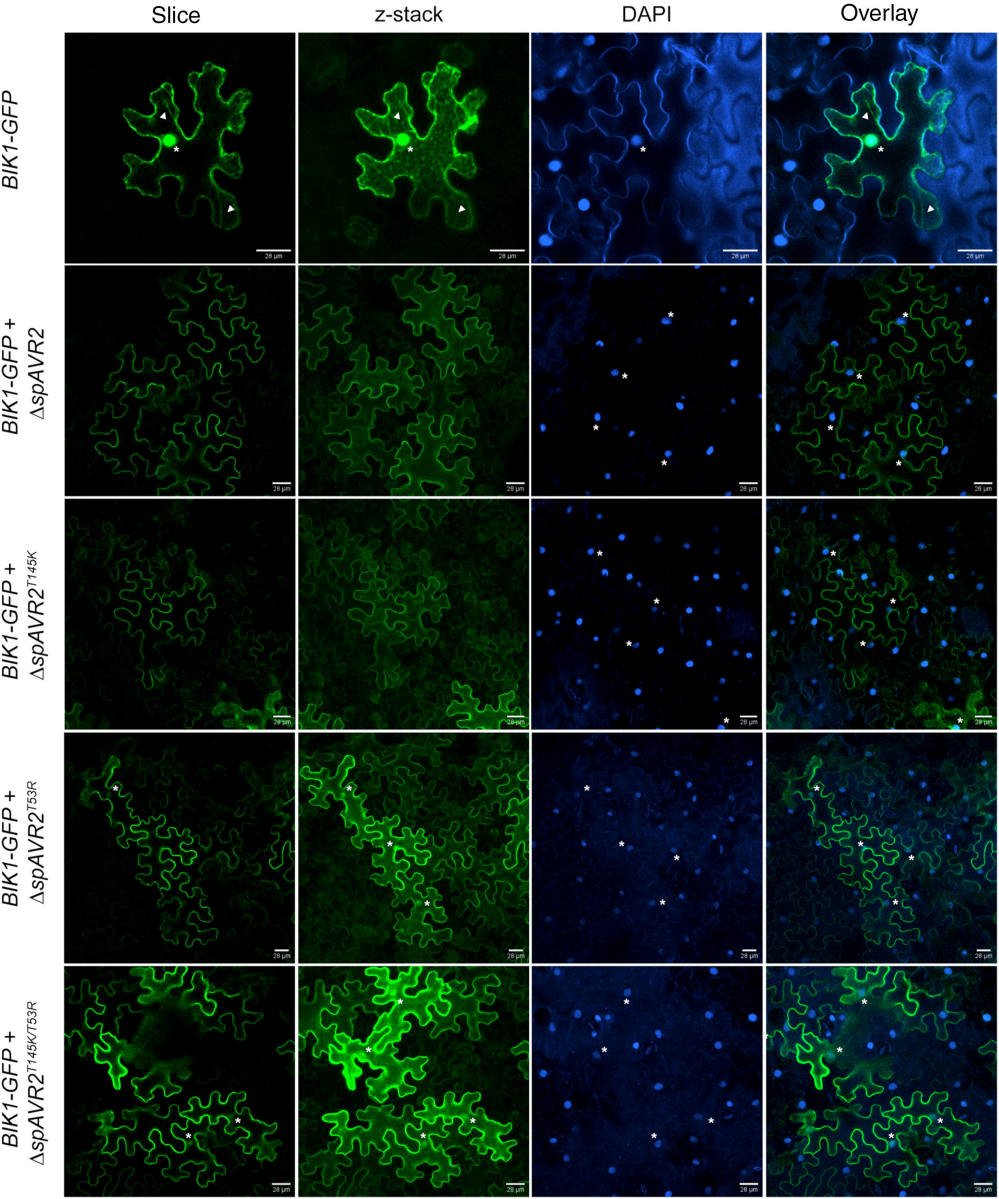
The subcellular localization of BOTRYTIS‐INDUCED KINASE 1 (BIK1) is altered by the Avr2 variants. Confocal microscopy images of agro‐infiltrated *Nicotiana benthamiana* leaves transiently expressing *BIK1‐GFP* in the absence or presence of Δ*spAVR2*or Δ*spAVR2T145K*, Δ*spAVR2T53R*, Δ*spAVR2T145K/T53R* variants. Asterisks mark green (GFP) or blue (DAPI)‐fluorescent signals located in the nucleus; arrowheads indicate green fluorescent signal located in cytosolic strands. Fluorescence was visualized 48 h after agro‐infiltration using confocal microscopy. Maximum projections of *z*‐stack images are depicted. Three biological replicates were performed, representative figures are shown. Scale bars represent 28 μm.

To assess the effect of the loss‐of‐virulence Avr2 mutants (Avr2T53R, Avr2T145K, and Avr2T145K/T53R) on the subcellular localization pattern of BIK1, we co‐infiltrated binary vectors containing either *BIK1‐GFP* and/or Δ*spAvr2*, Δ*spAvr2T53R*, Δ*spAvr2T145K*, or Δ*spAvr2T145K/T53R* in *N. benthamiana* leaves. A DAPI staining was included to accentuate the nuclei. As previously observed, in the absence of Avr2, green fluorescence originating from BIK1‐GFP was observed in the nucleus, cytosolic strands, and at the cell periphery (Figure 7, first row, indicated by stars and arrowheads, respectively). In the presence of Avr2, green fluorescence was found predominantly at the cell periphery and not in the nucleus nor in cytosolic strands (Figure 7, second row). Avr2T145K showed a BIK1‐GFP cell peripheral localization pattern like that of wild‐type Avr2 (Figure 7, third row). Interestingly, Avr2T53R and Avr2T145K/T53R permitted BIK1 mobility based on the weak green fluorescent signal in the nucleus (Figure 7, fourth and fifth rows).

## 3 DISCUSSION

This study investigates how Avr2 from the tomato root‐ and xylem‐colonizing strain of *F. oxysporum* suppresses immune signalling induced by a multitude of PAMPs. We show that in the presence of flg22 and Avr2 (i) the PRR FLS2 still dimerizes with its co‐receptor BAK1, implying that PAMP perception and the initial signalling steps are unaffected by Avr2, (ii) BIK1 is phosphorylated, demonstrating that the FLS2‐BAK1 complex is still functional, but (iii) mono‐ubiquitination of BIK1 is reduced and its accumulation and subcellular localization altered, suggesting retention of BIK1 at the PM as a possible explanation for the impaired signalling ability of this RLCK.

RLCKs are common targets for bacterial effectors to disrupt PTI signalling. For example, *P. syringae* effector AvrPphB, a cysteine protease, cleaves several RLCKs (PBS1, BIK1, PBL1, and PBL2) (Zhang et al., 2010). The structure of Avr2 resembles that of the TRAFdomain–containing proteins (Di, Cao, et al., 2017) that are known to interact with E3 ubiquitin ligases important for the addition of ubiquitin. Ubiquitination targets proteins for degradation or shifts their subcellular localization (Gao et al., 2022). Given this structural homology between Avr2 and TRAF domain–containing proteins, we hypothesize that Avr2 could target BIK1 function by interfering with its mono‐ubiquitination process to retain BIK1 at the PM.

The immune‐regulatory function of BIK1 depends on both its nuclear and cytosolic localization. Nuclear localization of BIK1 is required for activation of defence gene expression, for example in *A. thaliana* by phosphorylation of WRKY transcription factors (WRKY 33, WRKY50, and WKRY57) that are involved in regulation of the defence hormones salicylic acid and jasmonic acid (Lal et al., 2018). A cytoplasmic localization of BIK1, on dissociation from the immune receptor complex at the PM, is required, for example, for initiation of ROS signalling through phosphorylation of the PM‐localized *A. thaliana* RBOHD complex (Kadota et al., 2014; Li et al., 2014). In *Avr2*expressing tomato and *A. thaliana* plants the ROS burst is decreased on treatment with flg22, chitosan, or nlp24 (Di, Cao, et al., 2017; Tintor et al., 2020), consistent with the hypothesis that BIK1 can no longer activate the RBOHD complex. Notably, in the presence of Avr2 the localization pattern of BIK1‐GFP shifted from a nucleocytoplasmic localization to the PM/cell periphery. In the presence of Avr2 the BIK1‐GFP signal was no longer observed in the nucleus nor in cytosolic strands. An impaired ability of BIK1 to translocate to the nucleus to activate transcription factors corresponds with the reduced induction of PTI responsive genes in an *Avr2* transgenic tomato plant (Di, Gomila, et al., 2017). The Avr2 T53R and T53R/T145K mutants did permit BIK1 to enter the nucleus, consistent with the inability of these Avr2 variants to suppress PTI responses on PAMP treatment. Surprisingly, in the presence of the Avr2 T145K mutant, no nuclear entry of BIK1 was observed while this effector mutant is compromised in its PTI suppressing activity. It is tempting to speculate that a minor fraction of BIK1, below the detection threshold of our experimental setup, regained mobility sufficient to restore its signalling functions in the cytoplasm and nucleus. Taken together, these findings support a mechanism by which Avr2 prevents dissociation of (activated) BIK1 from the PM to allow activation of RBHOD to induce ROS production and defence gene expression.

Release of BIK1 from the PM requires several actions, starting with PAMP recognition by the FLS2–BAK1 complex followed by *trans*‐phosphorylation of BIK1 by BAK1 and subsequent mono‐ubiquitination of BIK1 by E3 ligases RHA3A/B (Ma et al., 2020). As heterodimerization of the FLS2–BAK1 complex was unaffected in *Avr2*‐overexpressing *A. thaliana*, the posttranslational modification of BIK1 seems a more likely process to be targeted by Avr2. Phosphorylation of BIK1 was still observed in the presence of Avr2, indicating that phosphorylation sites of BIK1 were readily accessible and functional. Mono‐ubiquitination of BIK1, however, was found to be reduced by Avr2, although not completely abolished. A pool of mono‐ubiquitinated BIK1 was still detected in the presence of Avr2, which is in line with Avr2 not being able to completely block PTI defence responses (Di, Cao, et al., 2017). Because mono‐ubiquitination of BIK1 is required for its release from the membrane complex, interference with this process by Avr2 would provide an explanation for the observed BIK1 retention at the cell periphery/PM. The mechanism by which Avr2 differentially affects BIK1 accumulation in *A. thaliana* and *N. benthamiana* is unclear; possibly, retention of the kinase at the PM affects endocytosis and protein turnover. Whether the observed difference is dependent on the plant species or the expression method awaits generation of stable transgenic *N. benthamiana* plants, and thus remains a question for future research.

How Avr2 may interfere with mono‐ubiquitination of BIK1 is unknown. *Xanthomonas campestris* pv. *campestris* type III effector AvrAC is a uridylyl transferase that targets two RLCKs, BIK1 and RIPK. By adding uridine 5′ monophosphate, conserved phosphorylation sites in the activation loops of BIK1 and RIPK are concealed, thereby reducing the kinase activity (Feng et al., 2012). Perhaps Avr2, which shares structural homology with TRAFdomain–containing proteins, masks the ubiquitination site of BIK1, thereby concealing it from the RHA3A/B E3 ligases. A possible competition of Avr2 with RHA3A/B for this binding site agrees with the incomplete repression of BIK1 mono‐ubiquitination and partial PTI suppression by Avr2. Avr2 point mutations T145K or T53R (located on opposite interfaces of Avr2 protein) confer loss of Avr2‐mediated PTI suppression (Di, Cao, et al., 2017). Interestingly, these Avr2 variants were still able to reconstitute fluorescence in BiFC assays with BIK1, implying their proximity to BIK1. Together with the compromised ability of the mutants to dimerize, it is tempting to speculate that the Avr2 dimerization interface is required for its PTI suppressing activity. Possibly this Avr2 interface is involved in recruitment of additional proteins such as the E3 ligases RHA3A/B. *X. campestris* pv*. vesicatoria* effector AvrBs3 homodimerizes prior to nuclear import. A specific repeat region in this effector is essential for virulence and self‐interaction (Gürlebeck et al., 2005). *Phytophthora sojae* effector PsCRN63 also forms homo‐ and heterodimers and dimerization is required for PTI suppression activities (Li et al., 2016). Likewise, dimerization of Avr2 seems to be important for the activity of Avr2 regarding PTI suppression.

Avr2 compromises ROS accumulation, callose deposition, and MAPK activity on PAMP treatment (Di, Cao, et al., 2017; Tintor et al., 2020). Because BIK1 does not regulate flg22‐, elf18‐, or chitin‐triggered MAPK activity (Feng et al., 2012; Yamada et al., 2016), it is likely that Avr2 also targets RLCKs besides BIK1. It is not uncommon for effector proteins to affect multiple RLCKs. *Xanthomonas* effector AvrAC, for example, targets both BIK1 and RIPK, while *Pseudomonas* effector AvrPphB targets as many as four RLCKs. Mutants of RLCK class VII‐4 (i.e., PBL19; PBL20; PBL37; PBL38; PBL39 [PCRK1], PBL40 [PCRK2]) are compromised in chitin‐triggered MAPK activation (Rao et al., 2018). For example, PBL19 was shown to phosphorylate MEKK1, resulting in MPK4 activation (Bi et al., 2018). Notably, PBL1 and PBL10, members of the RLCK class VII‐8 together with BIK1, are also subjected to mono‐ubiquitination in a PAMP‐dependent manner (Ma et al., 2020). MAPK activation, however, is not affected in a *bik1/pbl1* double mutant (Ranf et al., 2014). Hence, it is conceivable that Avr2 targets the function of multiple RLCKs involved in PTI signalling.

In conclusion, the effector Avr2 of the tomato root‐ and xylem‐colonizing strain of *F. oxysporum* interferes with PAMP‐induced mono‐ubiquitination of BIK1 and causes retention of this RLCK at the PM, compromising its ability to induce PTI responses. Although effector‐mediated PTI suppression has been well established for foliar pathogens, this report of an effector of a root‐colonizing pathogen that suppresses PTI through targeting a conserved signal transduction component is a novel find. The manipulation of immunity through PTI suppression is seemingly of importance to root‐infecting pathogens as well. Root‐based PTI defence responses are spatially restricted to tissues surrounding the vasculature (Emonet et al., 2021). It is therefore noteworthy that Avr2 can move via the symplast (Blekemolen et al., 2022; Cao et al., 2018), allowing it to suppress PTI in distal uninfected cells prior to fungal colonization. It will be interesting to investigate whether this is a shared feature for PTI‐suppressing effectors from root‐colonizing pathogens.

## 4 EXPERIMENTAL PROCEDURES

### 4.1 Generation of BIK1, Avr2 point mutations, and BiFC constructs

To generate pDONR:BIK1 without a stop codon, PCR was performed on the *A. thaliana* coding sequence of BIK1 (geneID: AT2G39660) using the following primer set: FP9732 (5′‐ACAAGTTTGTACAAAAAAGCAGGCTCCATGGGTTCTTGCTTCAGTTC‐3′)/FP9734 (5′‐ACTTTGTACAAGAGAAAGCTGGGTCCACAAGGTGCCTGCCAAAAG‐3′). pDONR:BIK1 without a stop codon was subsequently used for Gateway cloning (Invitrogen) together with pGWB451 to generate pGWB451:BIK1, via an LR reaction.

To generate a pDONR:Avr2T145K/T53R double mutant, pDONR:Avr2T145K and pDONR:Avr2T53R (previously described; Di, Cao, et al., 2017) were used as templates for quick change PCR using the following primer sets: FP6917 (5′‐GCATCCCAACTGATTTTGGCTGGACCTCGAG‐3′)/FP6918 (5′‐CTCGAGGTCCAGCCAAAATCAGTTGGGATGC‐3′) and FP6929 (5′‐TGCTGAAGCTCGTCCTAAATGAAGTAGAAGACGTGCGGCG‐3′)/FP6930 (5′‐CGCCGCACGTCTTCTACTTCATTTAGGACGAGCTTCAGCA‐3′). pDONR:Avr2T145K/T53R clones were verified by sequencing.

SCYN or SCYC N‐terminally tagged versions of Avr2, Avr2T145K, Avr2T53R, and Avr2T145K/T53R were generated through Gateway cloning (Invitrogen). Entry vectors pDONR:Avr2(wt), pDONR:Avr2T145K, pDONR:Avr2T53R, and pDONR:Avr2T145K/T53R were cloned into destination vector pDEST:SCYN(gw) or pDEST:SCYC(gw), via an LR reaction, to generate *SCYC‐*Δ*spAVR2*, *SCYC‐*Δ*spAVR2T145K*, *SCYC‐*Δ*spAVR2T53R*, *SCYC‐*Δ*spAVR2T145K/T53R*, and *SCYN‐*Δ*spAVR2*. To generate *BIK1‐VYN* and *BIK1‐VYC* constructs, entry vector pDONR:BIK1 without a stop was cloned into destination vectors pDEST:(gw)VYN or pDEST:(gw)VYC via an LR reaction. All clones were verified by sequencing.

### 4.2 *A. tumefaciens* (agro) transient transformation of *N. benthamiana* leaves

Agro‐infiltration of 5‐week‐old *N. benthamiana* plants was performed as described (Ma et al., 2012). Co‐infiltrations of *A. tumefaciens* GV3101 (pMP90) strains containing *BIK1‐GFP* and Δ*spAVR2* were carried out at an OD_600_ of 0.5 each. For the BiFC experiments, co‐infiltrations of *BIK1‐VYN* together with *SCYC‐*Δ*spAVR2*, *SCYC‐*Δ*spAVR2T145K*, *SCYC‐*Δ*spAVR2T53R*, or *SCYC‐*Δ*spAVR2T145K/T53R*, and *SCYN‐*Δ*spAVR2* together with *BIK1‐VYC*, *SCYC‐*Δ*spAVR2T145K*, *SCYC‐*Δ*spAVR2T53R*, or *SCYC‐*Δ*spAVR2T145K/T53R* were carried out at an OD_600_ of 0.5 each. The viral silencing suppressor p19 was co‐infiltrated along with the other constructs at an OD_600_ of 0.1. Leaves were either subjected to confocal microscopy 48 h after agro‐infiltration or harvested for protein isolation after a 10‐min flg22 treatment (100 nM). *N. benthamiana* plants were grown in a climate‐controlled greenhouse at 22°C, with 70% relative humidity, and under short day light conditions: 13 h dark/11 h light.

### 4.3 Confocal microscopy

Confocal microscopy was performed with a Nikon A1 microscope (Nikon Instruments Inc.). Excitation of GFP and split SUPERCYAN/VENUS fluorophores was carried out at 488 nm with an Ar‐ion laser and the signal emitted was selected using a 500–525/550 nm bandpass filter. SUPERCYAN and DAPI were excited at 405 nm with a diode laser and light signal emitted was selected with a 445/480–515 nm bandpass filter. DAPI staining was performed 1 h before imaging by syringe‐infiltration of the DAPI solution into the previously agro‐infiltrated leaf sectors.

### 4.4 Protein isolation and (co)‐immunoprecipitation

Three‐to‐four snap–frozen *N. benthamiana* leaf disks (diameter 5 mm) were ground in liquid nitrogen and the powder was resuspended in 200–300 μL of extraction buffer (50 mM Tris–HCl pH 7.5, 2% SDS, 5 mM dithiothreitol [DTT] and 1× protease inhibitor cocktail; Roche) and centrifuged at 4°C at 15,000 *g* for 30 min.

Seven‐week‐old snap‐frozen *A. thaliana* leaves were ground using a TissueLyser II (Qiagen) and 25 mg of powder was resuspended in 100 μL of extraction buffer (50 mM Tris–HCl pH 7.5, 2% SDS, 5 mM DTT and 1× protease inhibitor cocktail; Roche), and centrifuged at 4°C at 16,000 *g* for 25 min.

Proteins were separated by SDS‐PAGE (Bio‐Rad) using either 10% or 14% acrylamide gels and subsequently blotted on polyvinylidenedifluoride (PVDF) membranes using the semidry blotting method (Thermo Scientific Owl HEP‐1 system). Blots were probed with rabbit or rat monoclonal Avr2‐ (Ma et al., 2015), GFP‐, or BIK1‐antibodies (Chromotek; Agrisera) at a dilution of 1:2500 or 1:5000. Secondary goat‐anti‐rabbit or goat‐anti‐rat antibody (Pierce) was used at a dilution of 1:5000. The signal was visualized with an ECL kit (GE Healthcare, ECL prime of Thermo Scientific, Super Signal West Pico) according to the manufacturer's instructions and detected using a ChemiDoc MP imaging system (Bio‐Rad).

For testing FLS2‐BAK1 complex formation in different *A. thaliana* genotypes, seedlings were sterilized and sown on ½ × MS (Duchefa Biochemie) agar plates and grown for 4 days (16 h light, 8 h dark). Subsequently, 15–20 seedlings were transferred per well of a six‐well plate containing ½ × MS liquid medium and grown for another 10 days. One day before treatment the seedlings were transferred into a glass beaker containing sterile water. The next day, flg22 was added to a final concentration of 100 nM and incubated for 10 min before harvesting seedlings by flash freezing in liquid nitrogen. Proteins were isolated in 50 mM Tris–HCl pH 7.5, 150 mM NaCl, 10% glycerol, 5 mM DTT, 1% protease inhibitor cocktail (Sigma Aldrich), 2 mM Na_2_MoO_4_, 2.5 mM NaF, 1.5 mM activated Na_3_VO_4_, 1 mM phenylmethanesulfonyl fluoride [PMSF] and 1% IGEPAL. For immunoprecipitations α‐rabbit Trueblot agarose beads (eBioscience) coupled with α‐FLS2 antibodies were used and incubated with the crude extract for 2–3 h at 4°C. Subsequently, beads were washed three times with wash buffer (50 mM Tris–HCl pH 7.5, 150 mM NaCl, 1 mM PMSF, 0.5% IGEPAL) before adding Laemmli sample buffer and incubating for 10 min at 95°C. Analysis was carried out by SDS‐PAGE and western blots using α‐FLS2 α‐BAK1 (Chinchilla et al., 2007) and α‐HA (clone 3F10; Roche) antibodies.

*BIK1‐HA* and empty vector or Δ*spAVR2‐FLAG* were transfected into protoplasts that were subsequently incubated at room temperature for 12 h. After treatment with 200 nM flg22 for the indicated time, protoplasts were collected by centrifugation at 100 *g* for 2 min and lysed in 4 × SDS loading buffer (250 mM Tris–HCl, pH 6.8, 40% vol/vol glycerol, 4% wt/vol SDS, 0.1% wt/vol bromophenol, and 4% vol/vol β‐mercaptoethanol) by vortexing. BIK1 phosphorylation was detected by α‐HA antibody (1:2000 dilution; Roche) after protein separation on a 10% SDS‐PAGE gel. For ubiquitination assays, FLAG‐tagged UBQ (*FLAG–UBQ*) and *BIK‐HA* were co‐transfected with the empty vector as control or with Δ*spAVR2‐GFP* construct into protoplasts and then incubated at room temperature for 12 h. For co‐immunoprepcipitation assays, *BIK‐HA* was co‐transfected with the empty vector as control or with Δ*spAVR2‐FLAG* construct into protoplasts and then incubated at room temperature for 12 h.

After treatment with 200 nM flg22 for the indicated time, protoplasts were collected by centrifugation at 100 *g* for 2 min and lysed in 300 μL of IP buffer (20 mM Tris–HCl, pH 7.5, 100 mM NaCl, 1 mM EDTA, 10% vol/vol glycerol, 0.5% vol/vol Triton X‐100, and protease inhibitor cocktail; Sigma) by vortexing. After centrifugation at 10,000 *g* for 10 min at 4°C, 30 μL of supernatant was collected as input control.

Immunoprecipitation was performed with anti‐FLAG M2 magnetic beads (Sigma‐Aldrich) for 3 h at 4°C. Beads were collected by Invitrogen DynaMag‐2 Magnet (Invitrogen) and washed three times with washing buffer (20 mM Tris–HCl, pH 7.5, 100 mM NaCl, 1 mM EDTA, 0.1% vol/vol Triton X‐100) and once with 50 mM Tris–HCl, pH 7.5. The ubiquitinated BIK‐HA was detected by anti‐HA antibody (1:2000 dilution; Roche), ΔspAVR2‐GFP by anti‐GFP antibody (1:2000 dilution; Roche), and ΔspAVR2‐FLAG by anti‐FLAG antibody (1:2000 dilution). Secondary goat‐anti‐mouse antibody (Cell Signalling) was used at a dilution of 1:10,000. Coomassie brilliant blue (CBB) staining for RuBisCO (RBC) served as loading control.

## FUNDING INFORMATION

F.L.W.T. and M.C.B. were supported by the NWO‐Earth and Life Sciences funded VICI project no. 865.14.003. Z.L and L.S were supported by NIH (R35GM144275), NSF (IOS‐2049642), and the Robert A. Welch Foundation (A‐2122‐20220331). C.Z. was supported by the Gatsby Charitable Foundation, the University of Zürich, the European Research Council under the grant agreements 309858 (grants PHOSPHinnATE), and the Swiss National Science Foundation (grant no. 31003A_182625). M.S. was supported by a postdoctoral fellowship (STE 2448/1) from the Deutsche Forschungsgemeinschaft.

## Supporting information


**Figure S1.** Photographs of SCYNΔspAvr2‐SCYCΔspAvr2 bimolecular fluorescence complementation (BiFC) interactions.

**Figure S2.** Avr2 and BIK1 do not co‐precipitate together.

**Figure S3.** Immunoblot analysis depicting BIK1‐GFP accumulation.

## Data Availability

The data that support the findings of this study are available from the corresponding author upon reasonable request.
