# Supplementary figures and images for: The PTI‐suppressing Avr2 effector from Fusarium oxysporum suppresses mono‐ubiquitination and plasma membrane dissociation of BIK1
Source: Mol Plant Pathol. 2023 Jun 30;24(10):1273–86. doi: 10.1111/mpp.13369 (PMC10502843; doi:10.1111/mpp.13369)

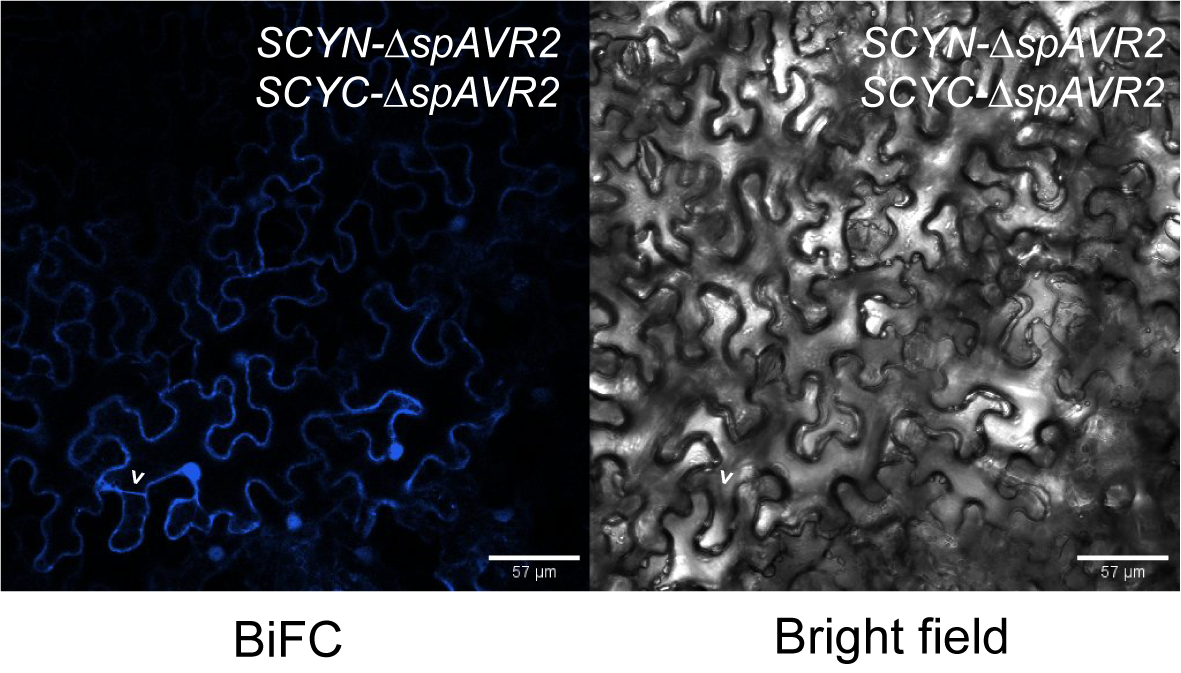

Supplement: Supplementary file 1 — Figure S1. Photographs of SCYNΔspAvr2‐SCYCΔspAvr2 bimolecular fluorescence complementation (BiFC) interactions. [file MPP-24-1273-s001.tif]

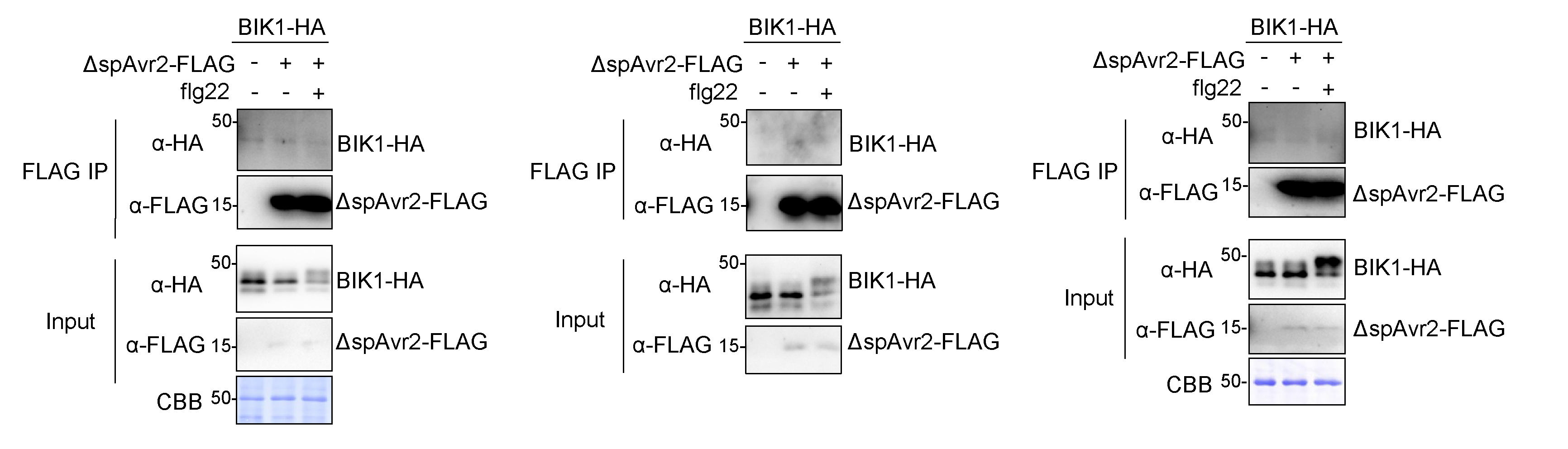

Supplement: Supplementary file 2 — Figure S2. Avr2 and BIK1 do not co‐precipitate together. [file MPP-24-1273-s003.tif]

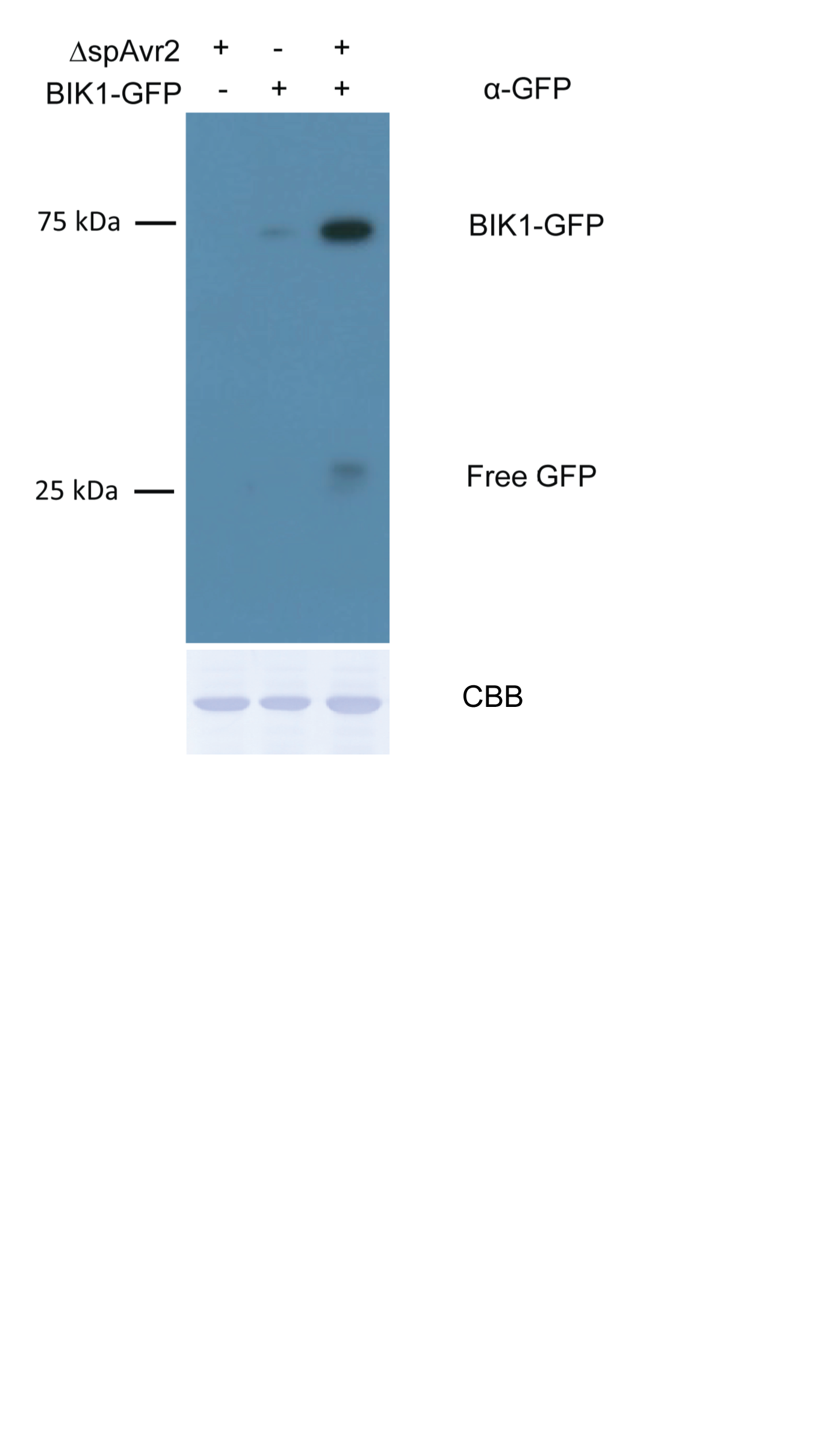

Supplement: Supplementary file 3 — Figure S3. Immunoblot analysis depicting BIK1‐GFP accumulation. [file MPP-24-1273-s002.tif]
